# Impact of an Alcohol-Based Hand Sanitizer Intervention on the Spread of Viruses in Homes

**DOI:** 10.1007/s12560-014-9141-9

**Published:** 2014-04-13

**Authors:** Akrum H. Tamimi, Sheri Carlino, Sarah Edmonds, Charles P. Gerba

**Affiliations:** 1 Department of Soil, Water and Environmental Science, The University of Arizona, Tucson, AZ 85721 USA; 2 GOJO Industries, Inc, Akron, OH 44311 USA

**Keywords:** Alcohol-based hand sanitizer, Hand hygiene, Virus transmission, Homes

## Abstract

The objectives of this study were to determine the movement of a virus throughout a household and the impact of an alcohol-based hand sanitizer (ABHS) on reducing the movement and exposure of the virus to household members. Bacterial virus MS-2 was used as the surrogate for human enteric and respiratory viruses. Seven households with families having at least two children in the age range of 2–18 living in the home were used in this study. The hands of one adult family member were contaminated with 1 × 10^8^. MS-2 bacteriophage in each home. After 8 h, the hands of each family member (10 fingers) and 20 frequently touched fomites were sampled to determine baseline contamination without intervention. Within 8 h, MS-2 was detected on all of the family member’s hands and most of the fomites. The intervention consisted of providing the families in all selected homes with bottles of an ABHS, which were placed in the kitchen, bathrooms, and nurseries. Smaller individual bottles were provided for each family member greater than 12 years old to place in purses, pockets, backpacks, etc. The families were instructed to use the ABHS one time or three times during the day. For one and three uses, a statistically significant reduction of virus on un-inoculated and inoculated hands of ~99 % occurred within 8 h. Similar reductions occurred on fomites throughout the households (97–99 %). These results demonstrate that the use of an ABHS can significantly reduce transfer of a virus to the hands, and to the commonly touched surfaces within the household.

## Introduction

Good hand hygiene involving hand washing and/or the use of alcohol-based hand sanitizers (ABHS) has been shown to reduce the risk of infection from both respiratory and enteric viruses (Prazuck et al. 2010; Stebbins et al. 2011, Warren-Gash et al. 2012). Most of these epidemiological studies have involved institutions or groups in developing countries. In a review of the impact of hand hygiene on the spread of respiratory infections Warren-Gash et al. (2012) concluded that their effectiveness varies depending on setting, context, and compliance. Epidemiological studies are costly, and confounding factors (e.g., multiple routes of transmission) and exposure models have been used to estimate the risks of infection and potential success of interventions (Nicas and Sun 2006; Nicas and Jones 2009; Zhao et al. 2012). While such models are useful to assess different interventions, they are not validated by experimental data.

Coliphage and virus DNA markers have been used to study the dispersion of viruses in indoor environments, such as day care centers, neonatal nurseries, and home settings. Rheinbaben et al. (2000) applied the coliphage ΦX174 to the hands of volunteers and doorknobs and then traced the spread of the virus to surfaces and other people in the home. Jiang et al. (1998) placed cauliflower virus DNA on objects in day care centers and found that it spread rapidly among toddlers. Another researcher placed the same viral DNA on telephones in a neonatal nursery hospital unit and found that it spread throughout the unit over a seven-day study period (Oelberg et al. 2000).

The objectives of this study were to study the movement of a virus throughout a household and the impact of an intervention with ABHS on reducing the movement and exposure of the virus to household members and fomites. Bacteriophage MS-2 has similar shape and size to many human disease-causing enteric and respiratory viruses likely to be spread in a home setting. Bacteriophage MS-2 infects the bacteria *Escherichia coli* and is very similar in shape and size (23 µm) to rhinovirus (common cold), norovirus (most common cause of adult gastroenteritis), and many other enteric viruses. For these reasons, it was selected as a surrogate.

## Methods and Materials

### Production of Phage

Bacteriophage MS-2 (ATCC 15597-B1) was assayed by the double-layer agar technique with *E. coli* ATCC 15597 as the host. The *E. coli* was grown overnight and transferred to Tryptic Soy Broth (TSB) (Difco, Sparks, MD) in a shaking water bath, and one ml of it was transferred to a new sterile broth for 3 h at 37 °C in a shaking water bath to reach the log growth phase. The virus was produced by collecting it from an infected lawn of *E. coli* by addition of 6 ml of TSB and then removing it with a pipette after 2 h. The suspension was then centrifuged at low speed to remove bacterial debris filtered through a 0.45-µm pore size membrane filter and finally stored at 4 °C until needed for further use.

### Experimental Protocol

To assess the spread of the MS-2 before the interventions, seven households with families having at least two children in the age range of 2–18 living in the home were selected randomly from a pool of available houses that were recruited. Basic demographic data for all households such as number of people living in the home, ages, sex, number and types of pets were obtained for all houses. The hands of one adult family member in each home were contaminated with 1 × 10^8^ MS-2 bacteriophage. After 8 h, the hands of each family member (10 fingers) and selected high-touch fomites shown in Table 1 were sampled to determine baseline contamination without intervention. These data are presented throughout this paper as “control” data.

**Table 1:** Fomites sampled

Room	Fomite
Kitchen	Fridge handle
	Kitchen counter
	Kitchen table
	Microwave handle
	Stove knobs
	Kitchen knobs
	Kitchen faucet
	Dishwasher
	Kitchen light switch
Bathrooms	Counters
	Faucets
	Door knobs
	Light switches
	Toilet flushers
Living rooms	TV remote controls
	Light switches
Bedrooms	Light switches
	Doors
Phones	Cell phones

In all phases, virus was added to the hands of one adult in the household. All individuals received one mL of a physiological saline suspension onto the palm of their hand; however, the virus was only applied to the hands of one adult member of the household. This was done so the household members did not know whose hand was inoculated. Each individual was then asked to gently rub the palm of one hand together with the other hand and move the hands in an upward motion so that the fingertips of one hand slide onto the palm of the opposite hand to disperse the virus onto the fingertips. The contamination of the adult hand occurred at approximately 8:30 AM in the morning. The family was asked to stay at home during the day and go about normal daily activities in between the sampling times. All studies were done on weekend days when families spent most of the day at home. During the study, family members were not allowed to use any other antimicrobial hand products including wipes, sanitizers, and disinfecting cleansers (bleach, disinfecting wipes, etc.). All families were asked to practice their normal hand washing routine using soap and water during the course of this study. The study consisted of a baseline phase, and then two intervention phases.

### Intervention

Families in all the selected houses were given 354 ml bottles of alcohol-based hand sanitizer (Purell Advanced Instant Hand Sanitizer, GOJO Industries, Akron, OH) to place in the kitchen, bathrooms, and nurseries (3–5 bottles depending on the household size). This product has an active ingredient of 70 % ethanol. Individual bottles (56 ml) for each family member more than 12 years old were provided to place in purses, pockets, backpacks, etc. Instructions on when and how to use sanitizers were given to the families. They were instructed to use the hand sanitizer three times during the day (8 h) in the first intervention phase, and once per day in the second intervention phase (Table 2). It was also recommended to apply enough sanitizer to keep hands wet for 15–20 s and to rub in thoroughly until dry. Baseline and Phase 1 (3 times per day use) were randomized; Phase 2 with only 1 use per day was always done last and was done with a subset of homes; and only 4 households were evaluated (Table 2). There was a minimum of one week between all study phases, and within 5 days after completion of a phase, all fomites sampled in the home were disinfected with a Clorox Chorine Disinfecting wipe (Clorox Company, Pleasanton, CA) to inactivate any viruses on the fomites. We have found these wipes to inactivate MS-2 virus.

**Table 2:** Experimental phases

Control phase No hand sanitizer. Hands and fomites sampled after 8 h
Phase 1 Hand sanitizer used 3 times during 8 h
Phase 2 Hand sanitizer used 1 time during 8 h

Individuals followed their normal hand washing routine during all phases of the study

The hands of the same adult family member in each home were contaminated with 1 × 10^8^ of MS-2 bacteriophage for both study phases. Eight hours after the adult hand was contaminated, the fingers (all 10 with a sponge stick) of each family member and fomites were sampled. The fomites sampled in each household are shown in Table 1. Fingers and fomites were sampled using a sponge stick (3 M, St. Paul, MN) containing Letheen broth. Approximately 100 cm^2^ of each fomite was sampled.

Permission for conduct of the study was obtained from the University of Arizona’s Office for Human Subjects Research prior to the study.

### Statistical Methods

Bacteriophage MS-2 concentrations were transformed to log base 10 to normalize the data. Geometric means were calculated for control and intervention on both hands and fomites. Average log reductions due to intervention were calculated based on geometric means. A 95 % confidence interval (CI) was constructed for the log base 10 reductions with upper and lower limits using student *t* statistic (Ott and Longnecker 2001). Percent reduction of MS-2 concentration was also calculated based on the geometric means. Analysis of Variance (ANOVA) was conducted using the R Language Libraries for each phase between the control and the intervention to determine statistically significant differences based on a rejection region of 5 % (Kabacoff 2011; Zumel and Mount 2013). An *F* statistic was calculated based on control and intervention MS-2 concentrations and was compared with the *F* value obtained for the 5 % rejection region. A *p* value was calculated to decide on the significant difference question. Bootstrapping ANOVA techniques present in the R Language were utilized whenever the conditions for the Classical ANOVA methods were not met (Kabacoff 2011; Zumel and Mount 2013).

## Results

Virus was detected on the hands of every member of the household, inoculated or non-inoculated, and on almost every fomite in the control (baseline) group. The only sites that were not contaminated in the control (baseline) period were toilet handles in two houses and bedroom light switches in three houses. The highest average virus concentrations for fomites were found in the kitchen and living room areas, as well as on cell phones. Reduction in virus on hands (inoculated and non-inoculated) and fomites with use of the ABHS was statistically significant (*p* < 0.0005) for Phase 1 (Table 3) and Phase 2 (Table 4). In addition, there was a drop in the total number of sites that were contaminated from the control to intervention. The percents of sites that were contaminated in the control (baseline) for Phase 1 and 2 was 97.98 and 97.12 %, respectively, and after intervention contaminated sites dropped to 64.92 % in Phase 1 and to 51.80 % in Phase 2.

**Table 3:** Impact of phase 1 (3 ×/day) intervention on virus occurrence on hands and fomites

Area sampled	Control geometric mean recovery	Post-intervention geometric mean recovery	*N*	Log_10_ reduction ± standard deviation	% reduction	*p* value
Hands (not inoculated)	1,102	12	25	1.97 ± 0.93	98.93	<0.0005
Inoculated hands	13,543	38	7	2.55 ± 0.96	99.72	<0.0005
Bathroom	285	7	64	1.61 ± 1.31	97.53	<0.0005
Bedroom	177	4	36	1.60 ± 1.45	97.46	<0.0005
Living room	1,080	8	13	2.13 ± 0.97	99.32	<0.0005
Kitchen	1,316	12	56	2.17 ± 1.27	99.27	<0.0005
Cell phones	1,316	24	19	1.74 ± 1.27	98.18	<0.0005
All fomites	646	10	216	1.81 ± 1.30	98.47	<0.0005
All fomites and non-inoculated hands	683	10	241	1.83 ± 1.27	98.52	<0.0005

**Table 4:** Impact of Phase 2 (1 ×/day) intervention on virus occurrence on hands and fomites

Area sampled	Control geometric mean recovery	Post-intervention geometric mean recovery	*N*	Log_10_ reduction ± standard deviation	% reduction	*p* value
Hands (not inoculated)	1,097	3	14	2.51 ± 0.99	99.69	<0.0005
Inoculated hands	10,255	16	4	2.81 ± 0.87	99.84	<0.0005
Bathroom	331	3	36	2.02 ± 1.31	99.03	<0.0005
Bedroom	58	2	18	1.45 ± 1.29	96.45	<0.0005
Living room	1,512	4	7	2.57 ± 1.76	99.73	<0.0005
Kitchen	1,531	5	32	2.48 ± 1.26	99.67	<0.0005
Cell phones	1,569	15	10	2.01 ± 1.73	99.02	<0.0005
All fomites	576	4	121	2.14 ± 1.36	99.28	<0.0005
All fomites and non-inoculated hands	615	4	135	2.18 ± 1.33	99.34	<0.0005

## Discussion

Enteric and respiratory viral infections can spread rapidly though households. Viruses can spread both from person to person and via fomites by hand contact (Boone and Gerba 2007). Exposure via contaminated fomites is common to influenza virus in homes with infected children. Boone and Gerba (2005) found 53 % of commonly touched surfaces were contaminated with influenza virus in homes with children that had influenza. The results of our study show that the presence of virus on just one person’s hands could result in the contamination of nearly all the fomite surfaces we tested within 8 h. Every room in the household became contaminated. Somewhat greater contamination occurred in the kitchen and living room, probably reflecting greater activity among household members in these areas of the house. The MS-2 virus also spread to the hands of all the other members of the household within that same period of time. MS-2 shows little decrease in titer within 30 min on hands and persists for days on surfaces such as stainless steel, Formica, and glass (Lopez 2012). The use of the ABHS was found to be effective in inactivating more than 99.99 % of virus when applied to contaminated hands (data not shown).

The use of the ABHS both once and three times a day was found to reduce the concentration of viruses on both the hands and commonly touched fomites by ~99 % in the studied households. In addition, when used three times per day, virus could no longer be detected on about half of fomites, indicating the use of ABHS completely stopped the transfer of the virus to those surfaces. Virus concentration on both hands and fomites were close to the detection limit of the assay (one viral PFU per hand or fomite surface sampled) and reductions may have been greater if a higher virus concentration had been used to contaminate the hand. This may also explain why the reduction in virus for one use per day was similar to three uses per day; if one use per day lowers the virus concentration to or just above the limit of detection, then the study is unable to detect a greater difference. Also the study was based on a single contamination event which is more representative of a person that contaminated their hands outside the home, and is not sick. It is not representative of the contamination that would occur if there was a sick person in the household, as they would be recontaminating the household continuously. Therefore, there would be a higher overall viral concentration in the household, and it is likely that a difference between one use and three uses per day could be detected. Future studies should evaluate the impact of ABHS in reducing viral concentration on hands and fomites where multiple contamination events occur.

A concentration of 10^8^ MS-2 was added to the hands to ensure enough viruses to trace its movement through the household. Although this may be high, this level of virus on the hands may not be unexpected. Norovirus, adenovirus, and rotavirus occur in concentrations up to 10^11^ to 10^12^ per g; thus, as little as 0.001–0.0001 g of feces could contain 10^8^ of these viruses (Haas et al. 1999). Thus, the seeded level of virus used in this study is in the realm of reality.

The results of this study demonstrate that the use of an ABHS can greatly reduce the exposure of family members to viruses in the household. The information generated in this study will be useful to validate models for the spread of viruses within households.
